# Seed Priming: A Feasible Strategy to Enhance Drought Tolerance in Crop Plants

**DOI:** 10.3390/ijms21218258

**Published:** 2020-11-04

**Authors:** Vishvanathan Marthandan, Rathnavel Geetha, Karunanandham Kumutha, Vellaichamy Gandhimeyyan Renganathan, Adhimoolam Karthikeyan, Jegadeesan Ramalingam

**Affiliations:** 1 Department of Biotechnology, Center of Excellence in Innovations, Agricultural College and Research Institute, Tamil Nadu Agricultural University, Madurai 625104, Tamil Nadu, India; vishvamarthandan56@gmail.com (V.M.); vgrenga@gmail.com (V.G.R.); karthick2373@gmail.com (A.K.); 2 Department of Seed Science and Technology, Agricultural College and Research Institute, Tamil Nadu Agricultural University, Madurai 625104, Tamil Nadu, India; seedmdu@rediffmail.com; 3 Department of Agricultural Microbiology, Agricultural College and Research Institute, Tamil Nadu Agricultural University, Madurai 625104, Tamil Nadu, India; kkumuthatnau@gmail.com

**Keywords:** drought stress, germination, hydration, seed priming, stress tolerance

## Abstract

Drought is a serious threat to the farming community, biasing the crop productivity in arid and semi-arid regions of the world. Drought adversely affects seed germination, plant growth, and development via non-normal physiological processes. Plants generally acclimatize to drought stress through various tolerance mechanisms, but the changes in global climate and modern agricultural systems have further worsened the crop productivity. In order to increase the production and productivity, several strategies such as the breeding of tolerant varieties and exogenous application of growth regulators, osmoprotectants, and plant mineral nutrients are followed to mitigate the effects of drought stress. Nevertheless, the complex nature of drought stress makes these strategies ineffective in benefiting the farming community. Seed priming is an alternative, low-cost, and feasible technique, which can improve drought stress tolerance through enhanced and advanced seed germination. Primed seeds can retain the memory of previous stress and enable protection against oxidative stress through earlier activation of the cellular defense mechanism, reduced imbibition time, upsurge of germination promoters, and osmotic regulation. However, a better understanding of the metabolic events during the priming treatment is needed to use this technology in a more efficient way. Interestingly, the review highlights the morphological, physiological, biochemical, and molecular responses of seed priming for enhancing the drought tolerance in crop plants. Furthermore, the challenges and opportunities associated with various priming methods are also addressed side-by-side to enable the use of this simple and cost-efficient technique in a more efficient manner.

## 1. Introduction

Drought is considered as one of the most destructive abiotic stresses across the world and creates a huge impact on crop production. The uncertainty of the global climate with erratic rainfall patterns is the major causes of the frequent onset of drought stress around the world [1]. Drought-induced economic losses were estimated to be about 29 billion dollars during 2005 to 2015, and it is predicted to become more persistent and extensive in the coming decades [2,3]. By 2050, about 50% of arable lands are expected to be under drought stress [4]. Drought can occur in all growth stages, but the first and foremost effect is on seed germination [5,6], where water entrance into the seed decreases due to hydraulic reduction; and thereby, all the physiological and metabolic germination processes are affected [7]. Impaired germination and establishment under drought stress have been studied in several crops viz., peas [8], barnyard millet [9], rice [10], and sunflower [11]. Inadequate supply of water for longer periods, affects the yield and productivity through negative impacts on phenology, growth, and reproduction [12,13]. Under severe water deficit conditions, cell division, elongation, and expansion were inhibited due to poor water flow from the xylem to the surrounding cells, which results in reduced plant height, leaf area, stem extension, and root proliferation [14]. Moreover, drought also induces the production of reactive oxygen species (ROS), such as superoxide radicals (O_2_^–^), hydroxyl radicals (OH^–^), and hydrogen peroxide (H_2_O_2_), which increases the lipid peroxidation and membrane deterioration and also affects the functions of biomolecules in the plants [15,16].

Plants generally acclimatize to survive under drought stress through the induction of various morphological (escape, avoidance, and phenotype plasticity), physiological (osmotic adjustment and cell membrane stability), biochemical (proline, auxins, and ethylene), and molecular mechanisms (stress-responsive proteins, transcription factors, and secondary messengers) [17,18,19]. However, the stress response varies with species, growth stage, and other environmental factors [20]. To cope with drought stress, the breeding of tolerant varieties; exogenous application of growth regulators, osmoprotectants, and plant mineral nutrients; and alteration in cropping patterns; etc. are being followed [21,22]. However, most of these practices are highly technical and the breeding of drought tolerant varieties is quite difficult due to the complex genetic nature of drought stress, inadequate knowledge on drought responsive genes/QTLs, and involvement of multidimensional stresses [23]. In recent years, various biotechnological approaches such as QTL mapping, characterization of drought-responsive genes, genome-wide association studies (GWAS), and genetic engineering are being followed to mitigate the effects of drought stress, but the risk assessors still face challenges in assessing the food and environmental safety of genetically modified crops (GM crops) [24]. On the other hand, agronomic management strategies such as surface tillage, spraying of anti-transpirants, selection of water-use efficient (WUE) genotypes, and reducing the evaporation by mulching are considered as the static tools in managing the drought stress, but these practices increase the cost of cultivation and are often inadequate on reconsideration for controlling crop performance [25].

Seed priming is a pre-germinative enhancement technique that will induce the early emergence of seedlings through the regulation of metabolic processes in the early phases of germination under drought stress [26]. Seed priming ensures increased and uniform germination by reducing the imbibition time [27], increasing the pre-germinative enzyme activation, increasing metabolite production [28], repairing the damaged DNA [29], and regulating osmosis. There are many reports on seed priming toward improving seed germination, seedling emergence, stand establishment, crop growth, nodulation, and productivity in various crop species viz., rice [30,31,32], wheat [33,34], pulses [35,36,37,38,39,40,41], okra [42], Chinese cabbage [43], sunflower [44], and melons [45]. Seed priming induces antioxidant activity and storage protein solubilization and minimizes lipid peroxidation [46]. Priming significantly increases the quantity of mitochondria and upregulation of proteins for cell division (α- and β-tubulin). Rehydration through seed priming brings major cellular changes in seeds such as de novo synthesis of nucleic acids and proteins; ATP (adenosine tri phosphate) production; activation of sterols and phospholipids; and repairing DNA damaged during threshing. However, the priming-induced molecular mechanisms are not studied as compared to transcriptome and proteome (omics) mechanisms behind the drought stress. Exploring the molecular mechanisms in the field of seed science may not only satisfy the seed traders but can also be useful for small and marginal farmers toward managing climate risk crop husbandry in a cost-effective manner [47]. Thus, the present review is intended to discuss (i) the impact of drought stress on seed germination and establishment, (ii) seed priming methods and their molecular mechanism of drought tolerance, (iii) challenges and opportunities, with the aim to promote the seed priming strategy as a future, cost-effective research tool to increase yield and productivity under drought stress.

## 2. Plant Responses to Drought Stress

**Figure 1:**
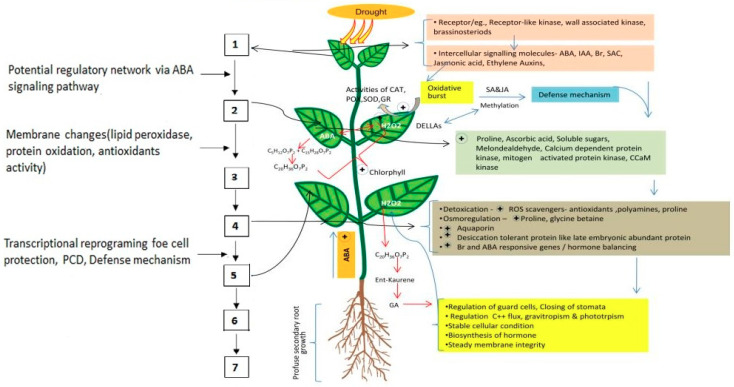
Schematic representation of drought response and stress tolerance mechanism. 1—Signal perception; 2—Cell signaling (signal transduction); 3—Transcriptional regulation; 4—Expression on regulation of stress responsive genes & cytoplasm protein transduction; 5—Respond to signal; 6—Cellular response; 7—Enhanced drought stress tolerance.

Plants express a dynamic response to sustain under stress conditions through morphological, physiological, and biochemical changes [48]. The response of plants to drought stress conditions has been categorized into drought escape, drought avoidance, and drought tolerance [49,50]. Drought escape in plants has evolved to regulate the growth period and to avoid osmotic stress in drought-prone areas [51]. Rapid growth, high photosynthetic capacity, high nitrogen level, and early flowering allows the plants to produce seeds before the onset of drought [50]. Drought avoidance operates through the regulation of a morphological and physiological phenomenon, which tends to save the plants from osmotic stress through elevated root growth, minimized stomatal counts and conductance, reduced leaf area, thickening of leaves, biosynthesis of cuticular wax on plant parts, and folding of leaves to minimize the evapotranspiration [52,53]. Drought tolerant plants maintain the osmotic pressure through cellular, biochemical, and osmotic alterations [54,55] and are capable of accumulating a variety of osmolytes (glycine betaine, proline, polyols sugars) in response to osmotic stress. These osmolytes protect the structure and integrity of biomolecules and membranes, and also act as free-radical scavengers from the damaging effects of ROS [56,57] (Figure 1).

### 2.1. Morphological Response

Successful plant growth under drought stress lies in the functional equilibrium between the root and shoot [58]. The root is the crucial component for sensing external stimuli and sends signals to the shoot to cope with drought stress [59]. Plants regulate their shoot/root ratios variably with the availability of substrates and environmental conditions [60]. An increased root:shoot ratio enables the plants to maximize the water uptake under drought stress [61]. Under water-limiting conditions, the shoots decrease their concentrations of sugars, amino acids, nucleosides, N, P, and K; on other hand, roots increase these components [62]. This results in reduced vegetative shoot growth, while the roots became more elongated and branched [63]. The water deficit condition has an effect on new leaf or branch formation, elongation, and expansion of stems and leaves and increases the production of ROS [64,65]. However, plants have developed several adaptive mechanisms, for example, leaf-rolling, (change in leaf angle to reduce the surface area exposed to sunlight) [66], reduced leaf area, epicuticular wax deposition, presence of awns, glaucousness, and hairiness are some of the important morphological drought-adaptive mechanisms to decrease transpiration and photosynthesis at the canopy level [67,68,69]. The water-use efficiency (WUE) is another mechanism of plant adaptation in the arid environment [70,71], where the tolerant genotypes have shown increased WUE with increase in biomass and also have deeper and denser roots than susceptible genotypes [72,73]. The plants such as thyme survive under drought stress by developing an extensive root system through reduced vegetative development [74].

### 2.2. Physiological Response

Generally, drought is mostly linked to changes in leaf anatomy and ultrastructure and causes the wilting of leaves, reduction in plant growth, decrease in the formation flower buds, and reduced leaves and leaf area [75]. Khosroshahi et al. reported that drought stress is a cause for the reduction of the plant organelles’ fresh weight and dry weight, number of leaves, total leaf area, relative water content, stomatal size, and frequency [76]. Photosynthesis is the first-line process that gets altered by drought stress [77]. Under water deficit conditions, the intercellular CO_2_ assimilation is altered due to the reduced activity of stomata via altered activities of CO_2_-fixing enzymes, membrane disruption, and reduced ATP synthesis, which ultimately inhibits the ribulose-1,5-bisphosphate carboxylase (RUBISCO) activity [78,79,80]. Moreover, the photochemical efficiency of photosystem (PS) II activity is also reduced due to disturbances in the electron transport mechanism, light-harvesting complex, structural and functional integrity of extrinsic polypeptides, and binding ability of ions (Ca and Mg) [81,82]. Reduction in relative water content (RWC) is the earliest effect of drought on plants; low relative water content reduces the leaf water potential and leads to the closure of stomata, which ultimately reduces the transpiration loss and increases the leaf temperature. Increased leaf temperature leads the disruption of the overall metabolic functions such as respiration, photosynthesis, ion and nutrient uptake, and synthesis of amino acid and proteins [83]. Non-availability of CO_2_ due to prolonged stomatal closure facilitates the accumulation of reduced ETC (electron transport chain) compounds; however, the accumulation of these compounds reduces the availability of molecular oxygen and increases the production of ROS species, resulting in oxidative injury to chloroplasts. In order to withstand drought, plants have adopted various adaptive mechanisms to protect themselves against desiccation [84,85]. A profuse root growth is advantageous to support crop growth during the initial growth stage to extract water from the shallow layers; otherwise this water is lost by evaporation [86]. Under water deficient conditions, root-induced signaling pathways to the shoot via the xylem induce physiological alterations to adapt under drought stress. During water deficit condition, the O_2_ supply around the root zone is depleted due to reduction in photorespiration, which affects the ATP production, eventually declining the ATP/ADP (adenosine di phosphate) ratio [87]. The plant senses the decrease in ATP levels and induces alcoholic fermentation with electron acceptors such as acetaldehyde and pyruvate by replacing oxygen. Similarly, when the plant is exposed to osmotic stress, the amount of chlorophyll pigment gets decreased, but this increases the rate of production of oxygen-containing carotenoid pigments (xanthophyll) [88] to have a role in protecting the plant from the adverse stress condition by inhibiting the ROS production [89]. Furthermore, the natural plant hormones also act as stress-responsive agents during the osmotic stress. Auxins plays an important role in plants under severe drought stress by restricting the growth of the plants [90]. In contrast, ethylene generates a negative response with abscisic acid (ABA) synthesis for the regulation of root and shoot development during drought [91]. Cytokinin also has a role during drought by stabilizing the photosynthetic apparatus, and exogenous application of cytokinin has been found to have a positive role under drought stress [92].

### 2.3. Biochemical Changes

Plants under prolonged drought experience a reduced net photosynthetic rate due to disturbances in biochemical processes due to the oxidation of chloroplast lipids and changes in the structure of pigments and proteins. Oxidative stress unbalances antioxidant defenses and ROS production causes the disruption of the cell membrane, degradation of proteins, and inactivation of enzymes [93]. In response to drought-induced oxidative stress, plants activate various antioxidants [28], such as catalase, superoxide dismutase, peroxidase, ascorbate peroxidase, glutathione, and ascorbate [94]. Similarly, abscisic acid (ABA) is a natural stress-responsive hormone, responsible for the expression of several transcription factors by encoding proteins for the synthesis of osmoprotectants [95]. It not only plays an important role in the plant life cycle as a hormone but also has an influence on several physiological processes to adjust with drought stress. Besides ABA, jasmonic acid, salicylic acid, brassinosteriod, cytokinins, and ethylene also take part in the stomatal stress response under extreme drought stress [96]. For instance, jasmonic acid is reported to be responsible for consistent ABA accumulation in the root system during water deficit conditions [97]. Similarly, the accumulation of proline and glycine betaine in cells under drought stress is also an important tolerance mechanism by adjusting the osmotic stress to maintain the structure and integrity of the cell wall [98,99]. Increased proline content under the water deficit condition is due to the production of complex molecules within living cells [100]. Proline is synthesized by two enzymes such as P5C (pyrroline-5-carboxylate) synthase and P5C reductase. Proline is assigned to regulate many physiological events, such as to maintain the stability of macromolecules, maintain the structural integrity membranes, and for scavenging the ROS [101]. Post-drought recovery is also possible through proline [98]. The reason for increased tolerance in transgenic plants is the huge production of P5C synthase, which accumulates osmoprotectants (proline, ornithine, and arginine) during drought [56]. Glycine betaine, on the other hand, has a protective role in the unfolding and denaturation of proteins through direct interaction with macromolecules or the formation of hydration shells around macromolecular complexes [102].

### 2.4. Molecular Changes

Molecular changes in response to drought stress include the production of drought-responsive genes (*DREB2* and *AQP7*), transcription factors [NF-Y (nuclear factor Y), ERF (ethylene responsive factor), and NAC (NAM—no apical meristem; ATAF—Arabidopsis transcription activation factor; CUC—cup-shaped cotyledon)], aquaporins, late embryogenesis abundant proteins, dehydrins, and heat shock proteins [103]. Drought triggers various molecular networks, secondary messengers (Ca^2+^, ROS, ABA, phosphoglycerol, diacylglycerol), and transcriptional regulators (protein kinase, protein phosphatase, and transcription factors) to adapt under stress [104,105]. The alterations in the gene expression of drought-affected plants induce a better response to drought stress [106,107]. The induction of these genes under stress is regulated through complex transcriptional networks such as abscisic acid (ABA)-dependent signaling pathway mediated by ABA-responsive cis-element-binding protein/ABA-responsive cis-element-binding transcription factors and ABA-independent signaling pathway mediated by dehydration responsive element-binding (DREB)-type transcription factors [108]. The synthesis of 9-cis-epoxycarotenoid dioxygenase (NCED), a key enzyme for ABA biosynthesis, is found to be increased during drought tolerance in *Arabidopsis* [109]. The synthesized ABA binds to the promoter region (ABA-responsive cis-element) to induce the production of drought-responsive genes [110]. On the other hand, DREB2 (DREB2A, DREB2B) proteins are important members of the AP2/ERF (apetala2/ethylene responsive factor) family of plant-specific transcriptional activators in the ABA-independent pathway. Transgenic plants overexpressing these two genes (*DREB2A, DREB2B*) have been found to have increased drought tolerance in rice [111,112]. Todaka et al. reported that, the genes responsible for drought tolerance are under intricate control as well as being species dependent [111]. For instance, higher expression levels of genes encoding isocitrate lyase and malate synthase in the glyoxylate cycle under abiotic stress condition were observed in rice but not in *Arabidopsis*. Lenka et al. have also identified the upregulation of α-linolenic acid metabolic pathway in the drought-tolerant genotype of rice [113]. Late embryogenesis abundant (LEA) proteins are important stress-inducible proteins involved in protective roles of cell membrane and protein by acting as cryoprotectants and osmoprotectants during water stress [114,115]. *HVA1*, is a LEA group protein gene, specifically accumulated in the aleurone layers and embryos of barley at the seed maturation stage, and was found to increase the tolerance to drought stress in transgenic rice plants [116]. Similarly, overexpression of *OsLEA3-1* and *OsLEA3-2* genes in rice also leads to enhanced drought tolerance [117,118].

Many researchers have reported that drought stress induces the abnormal expression of large miRNAs (micro RNAs); as a result, miRNAs may be a novel factor for the genetic modification of plants against the drought stress. The role of miRNA in abiotic stress tolerance was reported in various studies [119], similarly drought induces the expression of Hv-miR827 in barley [120] and the upregulation or downregulation of various miRNAs was identified in rice [121] under water deficit conditions. The regulation of gene expression at the transcriptome level is influenced by transcription factors (TFs) [122]. Several TF genes were identified from different families to change the network of gene expression for drought adaptation in plants, such as *MYB, MYC, NAC, bZIP, HD-ZIP,* and *DERB,* and these have gained attention due to their magnificent role in drought tolerance through ABA-independent or ABA-dependent pathways [123,124]. Overexpression of *AtWRKY57* hoists the ABA level and induces drought stress tolerance in *Arabidopsis*. Expression of *AtWRKY63* and *BdWRKY36* overcomes osmotic stress through the ABA signaling pathway in transgenic tobacco [125,126]. The overexpression of *ZmNAC111* in transgenic maize crops helps to improve WUE and enhance the expression of drought-responsive genes [127,128].

### 2.5. Metabolomic Changes

Metabolomic components are also a critical factor to induce drought tolerance [129]. The crop response to stress characteristically starts with a detailed signaling network, in addition with repeated crosstalk between the primary and secondary metabolic pathways [130]. When the plants adapt under drought stress, it does reorganize their metabolic pathways and promotes the upstream production of metabolites and downstream utilization of metabolites [131]. Susheela et al. reported that leaf protein gets altered by the drought stress by change in the fraction of soluble protein level in *Quercus rubra* [132]. Similarly, the levels of myo-insitol and galactinol are the precursors in the synthesis of raffinose family oligosaccharides (RFOs) during drought conditions [133]. RFOs interact with the phosphate group of the lipid membrane and macromolecules, altering the fluidity of cytoplasm for cell stabilization during drying via reversible cell vitrification [134].

The increase of amino acids such as valine, glutamine, ornithine, tryphotophan, and tyrosine is related to the accumulation of accessible substrate for the synthesis of proteins and faster recovery of plant metabolism after drought stress [135]. The extensins are a family of hydroxyproline-rich glycoproteins (HRGPs), enriched hydroxyproline provides sturdiness to the cell wall during drought stress conditions [136]. Ornithine is an intermediate component utilized for the biosynthesis of arginine [137]. Asparagine accumulation in vegetative tissues will occur in response to osmotic stress conditions [138]. Urea is an osmolyte, but at higher concentrations it will affect protein folding and protein binding. Urea is the best and greatest source of nitrogen-containing metabolites strongly associated with the degradation of protein and its level is increases in the drought-stressed leaves. Urea is synthesized in the mitochondria but stored in vacuoles in the purslane family, the higher concentration of urea accumulated in the vacuole helps to maintain the osmolarity [139].

## 3. Seed Priming

**Figure 2:**
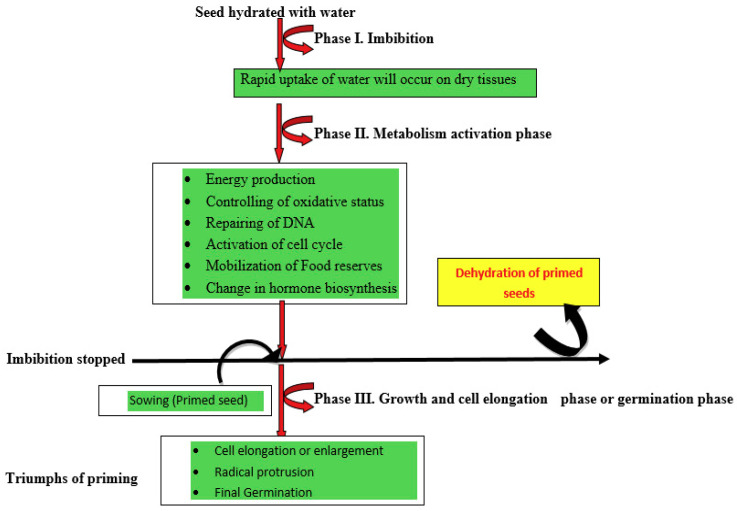
Thematic flow chart of sequence of events upon seed priming.

**Figure 3:**
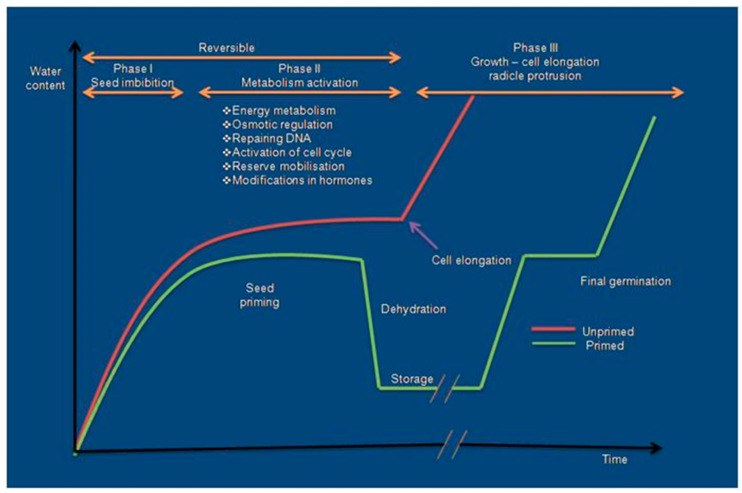
Seed imbibition curves and phases of seed germination in unprimed and primed seeds.

Seed priming is a pre-sowing, controlled hydration technique, which allows germination metabolic processes to proceed without actual germination [140] (Figure 2). Seed priming hydrates the seed to activate the pre-germinative metabolic and biochemical activities without radical protrusion during phase II of seed germination [141,142] (Figure 2 and Figure 3). The primed seeds facilitate uniform germination by enzyme activation, cell repairing mechanism, synthesis of proteins, and improved antioxidant defense mechanisms as compared to non-primed seeds [143,144]. Seed priming also enhances the accumulation of osmolytes (proline, glycine-betaine, and polyamines) through altered metabolic processes [145] and state transition from non-germinated to germinated and vice versa in response to the hydration of the seed. Different types of priming treatments such as water-based, PGR-based (plant growth regulator), osmotic solution-based, chemical-based, etc., are widely using to enhance the drought tolerance in many crop plants. Although all these techniques have a common feature, i.e., partial pre-hydration and earlier activation of germination events in seeds, the efficiency highly depends on the treated plant species and the chosen priming technique. The following Section 3.1, Section 3.2, Section 3.3, Section 3.4, Section 3.5, Section 3.6, Section 3.7 cover different seed priming methods and the mechanisms involved in enhancing the tolerance capability in crop plants.

### 3.1. Hydropriming

Hydropriming is a simple, low-cost, and environmentally friendly seed priming technique, which involves the soaking of seeds in normal water and dehydrating them to their original moisture content before sowing [146]. It augments moisture content with continuous oxygen supply and induces the accumulation of hydrolytic enzymes (amylase, cellulase, and xylanase) to convert stored food products (carbohydrates, proteins and lipids) into simpler forms (ATP) for pre-germinative metabolic processes [147]. Hydropriming effectively improved the seedling vigor, uniform germination, early seedling emergence, crop growth, and development in chickpea [148], faba bean [149], sweet basil [150], and rice [151] in drought stress condition compared to control [152]. Despite its advantages, uncontrolled water uptake by the seeds is the main disadvantage of this method, as the water uptake depends on the affinity of the seed tissue toward water. It is recommended to define accurate water volume, temperature, and duration for a desirable level of hydration to prevent radicle protrusion.

### 3.2. Osmopriming

Osmopriming is the most common priming method, which involves the hydration of seeds in a low-osmotic aerated solution with different time durations and water potentials [153]. The low water potential of osmotic solutions allows slow imbibition of water to activate pre-germinative metabolic processes without radicle protrusion. The commonly used osmotic solutions include KH_2_PO_4_, KNO_3_, CaCl_2_, MgSO_4_, NaCl, KCl, mannitol, and PEG (poly ethylene glycol), of which PEG is widely using in osmopriming [154]. The larger molecular weight of PEG restricts its diffusion into the seed to avoid cytotoxic effects and also lowers the solute potential. The controlled hydration in osmopriming reduces the production of ROS, since shortage of water during hydration creates stress to produce ROS [155]. This method has more advantages than hydropriming, since osmopriming results in earlier germination and seedling emergence and also better response to other stresses such as salt and chilling [156]. Osmopriming with PEG effectively improved the germination and fresh and dry weights of the plumule in the seeds of caraway (*Carum carvi* L. var. *annua*) [157].

### 3.3. Chemical Priming

Priming agents such as chitosan, choline, putrescine, paclobutrazol, ZnSO_4_, CuSO_4_, KH_2_PO_4_, and selenium are used in chemo-priming for improving growth performance and stress tolerance [158]. The chemical priming agents enhance drought tolerance by osmoprotection, detoxification, and protein and ionic homeostasis. The greater penetration capacity of chemical agents through the seed coat improves the nutrient uptake and WUE. Pre-sowing seed priming with butenolide has improved the seedling strength and emergence in pepper and salvia [159]. Seed priming with mannose has improved the drought tolerance by increasing the antioxidants levels, reducing oxidative injuries, and accumulating higher amounts of reducing sugars for osmotic regulation [160]. Effective priming agents such as SiO_2_, Ag, and ZnO can also be converted to nanoparticles and used for seed priming treatment to improve seed germination and vigor [161,162].

### 3.4. Biological Priming

Biopriming integrates seed imbibition with biologically active bacterial inoculants in the priming solution [163]. Plant growth promoting *Rhizobacteria* (PGPRs), biocontrol agents, and fungicides are added to the priming solution to improve germination and seedling vigor, synchronizing crop stand, growth, and yield parameters with their tolerance to biotic and abiotic stresses [164,165]. The most commonly used PGPRs include *Trichoderma*, *Pseudomonas*, *Azotobactor*, *Azospirillum*, and *Agrobacterium* to improve drought tolerance [166]. Biopriming with *Trichoderma* induces the production of plant growth hormones to improve drought tolerance [167]. Biopriming with *Trichoderma* enhanced the redox state of plants through the higher activity of L-phenylalanine ammonia-lyase and increased the root vigor under drought conditions. It improves drought tolerance by physiological protective mechanisms against the oxidative injury through production ROS scavengers and increased resistance to diseases through coat filming over the seed [168].

### 3.5. Hormonal Priming

Hormonal priming involves the hydration of seeds in an aerated solute medium of various plant growth promoting hormones such as abscisic acid, kinetin, SA (salicylic acid), GA_3_, and ascorbate. Hormonal priming induces the crop stand under high temperature and drought condition [169,170]. Seed priming with spermidine and polyamines is found to be more effective to induce drought tolerance in rice [171]. Seed priming with ABA acts as a growth regulator in the plant system under limited soil moisture conditions through the accumulation of osmoprotectants [172]. Seed priming with gibberellic acid improves rye seed germination and induces the production of antioxidants under drought stress conditions [173]. Seed priming with benzyladinine (BA) prior to sowing significantly induced soybean growth, root biomass, and conversion efficiency, and these effects show that BA reasonably induced drought tolerance in soybean [69].

### 3.6. Solid Matrix Priming

In solid matrix priming (SMP), the matrix potential of the priming solution is controlled during seed imbibitions with the addition of solid matrix substances that produce matrix forces to hold water and slowdown the solute uptake by seeds [149]. The slow imbibition of solute keeps the seed moist for a longer period to sustain under drought stress. However, solid matrix substances have lower bulk density and lower osmotic potential, and maximum water-holding capacity. Matrix priming has the potential to supply a good amount of O_2_ to the seeds at the time of the seed priming process. Grzesik reported that solid matrix priming improved the pre-germinative metabolism in *Helichrysum bracteatum* L. [174]. SMP improved the emergence and establishment of carrot [153] as well as the germination and vigor of soybean. Seed priming with chitosan at 5% soil moisture significantly improved the drought tolerance in pea [175]. Kubik et al. found that matrix priming in pepper and tomato seeds shown a significant improvement in seed germination [176]. Solid matrix conditioning might be a good alternative to improve the seed germination in various crops species, particularly in horticultural crops. This technique has proven significant results in many crop species; however, it still needs to be explored at a larger level.

### 3.7. Nutripriming

During the osmotic stress condition, plants require additional water and nutrients for germination and growth. Nutripriming is the recently developed strategy, to improve the available nutrient and water to the emerging plant seed priming with the addition of magnesium, zinc, and boron effectively improving the germination, growth and development, early flowering, early maturity, grain filling rate, and yield of several field crops [177,178,179,180]. Rehman et al. revealed that chickpea seeds primed with boron increased the overall productivity, this might be due to the effective fertilization and seed set influenced by boron [181]. Potassium is considered as a major nutrient, it also acts like a traffic policeman [182]. Likewise, chickpea seed primed with Zn has been found to improve the crop canopy, drought tolerance index, and yield attributes [183]. Priming the seeds with calcium is more effective and feasible for improving stress tolerance in various crops [33,144]. Calcium plays a significant role as a secondary messenger and induces the accumulation of osmolytes and antioxidants under stress conditions [184]. Additionally, nutripriming also increases the tolerance to several environmental stresses, and some of the nutrients are known to improve the antioxidant production in crop plants.

## 4. Mechanisms of Seed-Priming-Induced Drought Tolerance in Crop Plants

**Figure 4:**
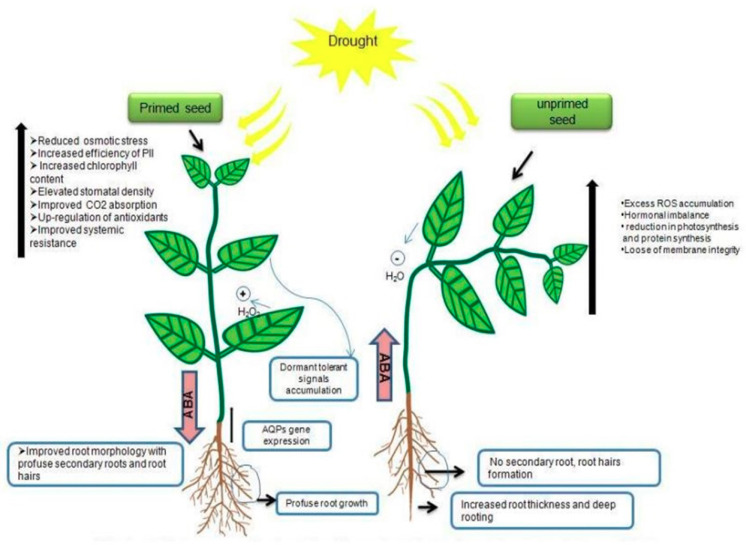
Performance of primed and unprimed seeds under drought condition.

Seed priming involves three different phases viz., (I) imbibition phase, where the rapid uptake of water will occur on dry tissues; (II) activation phase or lag phase, where the uptake of water is reduced and slight increase in fresh weight, rejuvenation of metabolic events, and repairing events at cellular level occur. The seeds are physiologically and metabolically very active in this phase, which helps in the development of mitochondria for ATP synthesis and synthesis of proteins from new mRNA and thereby, mobilization of the food material for radical growth. (III) Growth and cell elongation phase or germination phase, the germination gets started with resumption of the radical in addition with rapid water uptake [185] (Figure 3). Seeds are usually exposed to a variety of abiotic stresses in the initial stage of the germination process and, thus, oxidative damage of lipids, proteins, and nucleic acids is not an unusual event [186]. However, seed water uptake and the subsequent growth of the embryo are controlled by water transportation [187]. Seed priming induces rapid imbibition of seeds with a limited amount of water to start the pre-requisite metabolic events for pre-germination without radical protrusion. The priming process can extend the lag phase and also prevent the start of the log phase through restriction of the movement of water for completing the radical protrusion [188]. The hydration treatment allows the water movement up to 50% to reinitiate the metabolic events. Due to the rapid uptake of water, the induction of germination and the emergence and establishment of primed seeds occurs earlier compared to that of control seeds [189]. After seed priming treatment, the synchronized and earlier germination of seeds might be due to the decline of lag phase, increased enzyme activity, increased germination-promoting metabolites, and better osmotic regulation of primed seeds [180]. At a physiological state, seed priming induces various metabolic changes in the seed with the initiation of the imbibition process [142]. The rehydration of primed seeds brings major cellular changes in seeds such as de novo synthesis of nucleic acids and proteins, ATP synthesis, activation of sterols and phospholipids, and repairing of DNA. DNA repairing mechanism is an essential component of the pre-germinative metabolism; proper repairing of damaged DNA permits the embryo cells to restart the cell cycle and DNA replication [190]. Seed priming induces antioxidant activity and storage protein solubilization and minimizes lipid peroxidation [191]. A proteomic analysis of primed *Arabidopsis* seeds revealed the increase of various storage proteins and enzymes such as isocitrate lyase and amylases during germination [192]. Priming significantly increases the quantity of mitochondria and related proteins (α- and β-tubulin) that are responsible for cell division [26] (Figure 4).

According to [193], hydropriming of wheat seeds improved the WUE, which ultimately improved the yield. It was found that, hydropriming treatment hydrolyzing the endosperm tissues of *Solanum lycocarpum* and also enhancing the seed germination, seedling growth, and development by endo-β-mannanase activity, favored breaking the mechanical resistance for cell elongation in embryo and embryonic cover. The upregulation of xyloglucan endotransglucosylase during priming treatment was responsible for cell wall loosening and restructuring [194]. Yan et al. reported that rice seed primed with melatonin was observed under a transmission electron microscope and it was found that seed priming restored the integrity of the cellular structure [195]. Cytoskeleton restructuring is essential for cell elongation to promote radical emergence [196]. The primed seeds began cell division in advance to induce the accumulation of β-tubulin and to start the replication of DNA for early radical protrusion [189].

Seed priming reinforces the cellular defense response and improves the tolerance against biotic and abiotic stress through latent defense protein accumulation [196]. Aquaporins, a major intrinsic protein, regulate the water movement across the cell membrane via the intercellular region in both monocot and dicot families [197,198]. The deep understanding of the positive role of water on germination and the profile of aquaporin expression genes were studied during imbibition and primary embryo growth stages in many species such as *Arabidopsis* [199], *Brassica napus* [200], and *Oryza sativa* [201]. These studies confirmed the potential role of aquaporins on seed germination against abiotic stresses. Furthermore, the reduced rate of seed germination by silencing OsPIP1;3 (plasma membrane intrinsic proteins) and improved the germination by overexpression of OsPIP;3 under water deficit conditions. The experimental results concluded that OsPIP;3 is a prerequisite for obtaining the actual germination in rice. Ge et al. demonstrated that, the primed seeds of *Brassica napus* were studied during the event of germination, it was found that primed seed induced the expression of *BnPIP1* and had no effect on *Bn_γ_-TIP2* [201]. The transcription levels of *Bn-TIP1* and *Bn_γ_-TIP2* were found to be increased in primed seeds, but no such impact was found on unprimed seeds. The gene *BNPIP1* is required for water movement to turn on the storage nutrients and enzyme metabolism during germination in brassica seeds, whereas, *Bn_γ_-TIP2* expression depends on cell growth during radical protrusion. Seed priming induces the AP2/ERF transcription factors, which induce the production of secondary metabolites and, thus, improve the drought tolerance [202,203].

The seed priming methods induce the physiological mechanisms to increase yield of crops under drought stress [204]. Priming with chemical agents was found to be a better ameliorant for drought conditions. Recently several studies are focusing on exploiting the priming-induced drought tolerance mechanisms in different crops. Moreover, seed priming could also improve tolerance mechanisms to subsequent stresses, via the mechanism of “stress memory”, where the plant exposed to a certain stress can induce a stress memory for better and faster response in later stress events [205]. The plants formerly exposed to one type of stress (stress priming/hardening) may develop tolerance/protection to another kind of stress through the production of secondary metabolites [33,187]. Though, the time gap between the priming and subsequent stress is short, the molecular mechanism of seed-priming-induced drought stress tolerance to a subsequent stress persists for a longer time due to stress memory [206]. Post-translation modification (PMT) is a potential stress memory for priming the defense system, by activating the genes for stronger and faster transcription in drought stress response [207]. The induced expression of H3K4me3 (histone H3 lysine 4- trimethylation) and stalled RNAP II (RNA polymerase) are correlated with activation of *RD29B* and *RAB18*, signifying that histone modifications are involved in the drought priming memory in *Arabidopsis* [208]. Results of various studies reveal that drought priming could induce the tolerance mechanism at the jointing stage [209]; for example, tolerance to water deficit and heat stress conditions at the grain filling stage in wheat [210]. The progeny from drought-priming treatment own the stress tolerance mechanism through enhanced photosynthesis and increased antioxidant capacity, and this might have the potential to protect the plants from heat stress in wheat crop at the time of post-anthesis [211]. Similarly, osmoprimed and hydroprimed seeds showed improved transgenerational drought tolerance by modulating the water relations, osmolytes accumulation, malondialdehyde contents, and lipid peroxidation at the terminal growth stage [33]. Transgenerational memory of drought stress also effects the root and topology of the progeny in spring barley, for instance, the progeny from primed plants have a reduced shoot-to-root fraction and its leads to a reduction in root thickness compared to the progeny from control under drought conditions [212]. Priming before anthesis improved the tolerance to a succeeding drought stress during grain filling, resulting in enhanced photosynthesis rate, ascorbate peroxidase activity, and higher yield compared to non-primed plants of wheat [99].

Plant hormones also respond to drought by long distance signaling in root and shoot and organize the water transport. Among the plant hormones, ABA has decisive mechanism to induce tolerance against the drought [213]. Drought stress results in the production of a number of hormones, which could be involved in organizing the various physiological events as gesture molecules in signaling networks [214]. During water deficit conditions, the plants sense the stress by the sensors implicated in response signaling, and it can be transduced through various metabolic cycles through signaling pathways and transcriptional factors [215]. Seeds of *Arabidopsis* primed with the non-protein amino acid b-aminobutyric acid have shown tolerance against drought stress through ABA accumulation, the expression of stress-responsive genes, and the closing of stomata [216]. High ABA accumulation boosts cytosolic Ca^2+^ and activates the anion channel in the plasma membrane [217]. The parental line of rice treated with osmopriming agents helps in the accumulation of a high proline content through proline expression genes along with the removal of a methyl group from nucleotides in DNA in the preceding progeny under drought stress.

## 5. Research Gap and Future Perspectives

With the unpredictable environmental conditions and shrinking of cultivable land areas due to overwhelming urbanization, farming will be pushed to its brink. Water is the only factor that will decide the initial establishment, growth, and development of crop plants. The uncertainty of environmental factors has an impact on the growth and development of crop plants, in these cases we need to research whether the combined effect of stress on physiological, biochemical, and cellular changes may be overcome by the stress tolerance effects induced through seed priming. Seed priming is evolving as a simple, cost-effective technology to counteract the adverse effects of abiotic stresses. Most of the environmentally friendly priming agents are used to trigger natural defense mechanisms for stress mitigation and to increase crop yield and quality without any negative effects on crop plants. However, the treatment concentration and time duration differ for crop to crop and species to species. This is the major constraint of seed priming, since maximum number of trials are required to find the appropriate priming concentration and duration for each crop species. Till now, there are only a few reports pertaining to molecular expression of seed priming and no clear evidence to understand the molecular mechanism of priming responses in different crop species. This creates a huge gap to discharge the priming technique for commercial utilization. The focused research needs to be streamlined to determine the duration and solute concentration for priming. In certain cases, seed priming may cause a menace of medium contamination through microbes such as fungi and bacteria, this may seriously damage the seed germination. Therefore, a dedicated study is needed to address these issues. Although the effect of different priming agents against a variety of biotic and abiotic stresses were well proved, there are only a few reports on the mode of action of priming-induced drought tolerance. However, recent omics technologies have started to unravel the molecular mechanism of priming-induced drought tolerance by the priming agents. Investigating the effect of priming techniques through proteomic approaches is become the most promising part of applied research. Overcoming the poor storability of primed seeds still remains a big problem. A perfect storage technique needs to be developed for storing the primed seeds. Such inventions may revolutionize the farming in water-starved regions, where the farming societies are not aware of improved technologies.

Priming enhances the stress tolerance and improves performance of genotypes possessing QTL for stress tolerance in rice such as *Sub1* in Swarna *Sub1* for tolerance to water logging condition and *Pup1* in IR74 *Pup1* to improve phosphorous uptake. The integration of molecular approaches with seed priming may significantly improve the crop growth and productivity in challenging environmental conditions. The regulatory pathways that seems to have an impact on seeds through priming techniques need to be addressed in future. Recently, with the advancement of combinatorial chemistry, hundreds and thousands of compounds can now be easily screened for discovering novel bioactive molecules against abiotic stress.
